# Nurse-like cells promote CLL survival through LFA-3/CD2 interactions

**DOI:** 10.18632/oncotarget.13660

**Published:** 2016-11-26

**Authors:** Frédéric Boissard, Marie Tosolini, Laetitia Ligat, Anne Quillet-Mary, Frederic Lopez, Jean-Jacques Fournié, Loic Ysebaert, Mary Poupot

**Affiliations:** ^1^ CRCT UMR1037 INSERM-ERL 5294 CNRS-Université Toulouse III Paul Sabatier, Toulouse, France; ^2^ Pole Technologique CRCT, Plateau Imagerie, Toulouse, France; ^3^ IUCT-Oncopole, Toulouse, France

**Keywords:** tumor associated macrophages, cells cross-talk, cell survival, leukemia

## Abstract

In the tumoral micro-environment (TME) of chronic lymphocytic leukemia (CLL), nurse-like cells (NLC) are tumor-associated macrophages which play a critical role in the survival and chemoresistance of tumoral cells. This pro-survival activity is known to involve soluble factors, but few data are available on the relative role of cells cross-talk. Here, we used a transcriptome-based approach to systematically investigate the expression of various receptor/ligand pairs at the surface of NLC/CLL cells. Their relative contribution to CLL survival was assessed both by fluorescent microscopy to identify cellular interactions and by the use of functional tests to measure the impact of uncoupling these pairs with blocking monoclonal antibodies. We found for the first time that lymphocyte function-associated antigen 3 (LFA-3), expressed in CLL at significantly higher levels than in healthy donor B-cells, and CD2 expressed on NLC, were both key for the specific pro-survival signals delivered by NLC. Moreover, we found that NLC/CLL interactions induced the shedding of soluble LFA-3. Importantly, in an exploratory cohort of 60 CLL patients receiving frontline immunochemotherapy, increased levels of soluble LFA-3 were found to correlate with shorter overall survival. Altogether, these data suggest that LFA-3/CD2 interactions promote the survival of CLL cells in the tumor microenvironment.

## INTRODUCTION

Chronic lymphocytic leukemia (CLL) is characterized by an accumulation of monoclonal CD5^+^ mature B-cells in the lymphoid tissues, bone marrow and peripheral blood [1]. Before being released into the circulation, CLL cells divide in proliferation centers within lymph nodes, a microenvironment that is critical for tumor cell survival and chemoresistance [2–5]. This tumor microenvironment (TME) comprises extracellular matrix, chemokines, cytokines and non-malignant cells including CD4^+^ helper T cells [6], mesenchymal stromal cells [7] and monocyte-derived nurse-like cells (NLC) [8]. In CLL, NLC correspond to the tumor-associated macrophages (TAM) that are found in solid cancers [9, 10]. Like TAM, NLC mostly display an M2 phenotype with expression of the CD14, CD11b, CD68, HLA class II and CD163 markers [9–11]. In solid cancers, TAM promote tumor cell survival, either directly [12, 13] or through immunomodulation [14–16]. Moreover, TAM can induce tumor chemoresistance to cyclophosphamide, methotrexate, 5-flurouracile and paclitaxel [17–19]. In lymphomas, disease aggressiveness is associated with an increased frequency of lymphoma-associated macrophages [20]. Likewise, the progression of CLL is associated with lymph node infiltration by NLC [21]. *In vitro*, NLC can differentiate following long-term culture with peripheral blood mononuclear cells (PBMC) from CLL patients. Such NLC can prevent CLL cell apoptosis *in vitro* through the release of soluble CXCL12 [8, 22], BAFF or APRIL [23], or indirectly via stimulating the release of CCL3 or CCL4 by B cells [5]. However, soluble factors only partially protect CLL cells from apoptosis, therefore additional factors might also be involved.

As slightly raised in the literature, CLL/NLC contact could promote CLL cell survival *in vitro* [11, 23, 24]. Indeed, in follicular lymphoma contact between lymphoma cells and lymphoma-associated macrophages has been shown to support neoplasic B-cell growth [20, 25, 26]. In addition, in multiple myeloma contact with TAM protects tumor cells from spontaneous and chemotherapeutic apoptosis [27]. In CLL, there are conflicting reports that CD38 on the surface of CLL cells binds to NLC CD31 to allow CLL cell survival [24, 28]. Some ligand/receptor pairs have been found to be expressed by both normal B-cells/monocytes and CLL/NLC (e.g. BAFF/APRIL), questioning the specificity of such interactions to the TME. Whether and how any direct CLL/NLC cell interactions contribute to CLL cell survival also remains unclear.

Here we sought to address this issue. Our results show that, *in vitro*, CLL/NCL cell contact can prevent CLL cell apoptosis through a mechanism that involves the LFA-3 (lymphocyte function-associated antigen 3)/CD2 axis. Moreover, an exploratory cohort of 60 patients showed that soluble LFA-3 expressed at the cell surface of CLL cells correlated with increased overall survival after frontline rituximab-based immunochemotherapy. Thus, our data collectively support the targeting of the LFA-3/CD2 axis to specifically block pro-survival signals afforded by NLC, with few off-target effects against normal B-cells.

## RESULTS

### Active cross-talk between CLL cells and autologous NLC revealed by trogocytosis events

**Figure 1:**
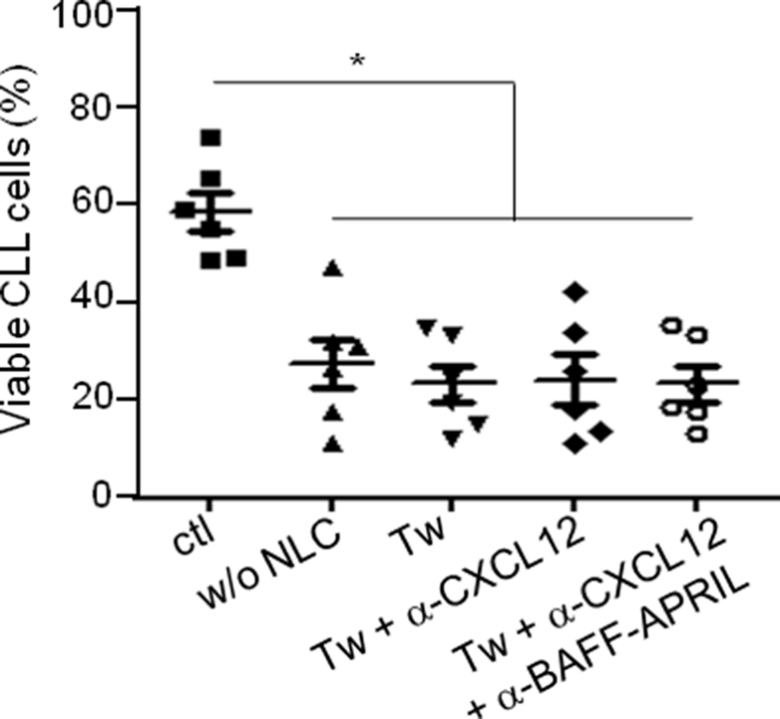
CLL viability is dependent on the contact with NLC Percentage viability of CLL cells after 7 days of culture in contact with NLC (ctl) or alone (w/o NLC) or separated from NLC by a Transwell membrane (Tw) in the presence or not of blocking antibodies against CXCL12 and/or BAFF-APRIL (6 independent experiments: 6 different CLL patients).

To confirm that direct contacts between CLL cells and autologous NLC are involved in the CLL survival, we performed co-cultures of CLL cells isolated from patients with autologous NLC separated or not by a Transwell membrane. The viability of CLL cells was evaluated with the ADAM-automatic fluorescent cell counter based on the propidium iodure staining. As expected, the culture of CLL cells alone without NLC induced a high decrease of the CLL viability (Figure 1). Moreover, separation of CLL cells from NLC by a Transwell membrane highly decreased the pro-survival activity gained from NLC co-culture (ctl condition). Then, we showed that the presence of antibodies against CXCL12, BAFF and APRIL did not amplify the CLL cells death in the Transwell conditions (Figure 1). This confirmed that NLC protect CLL cells from apoptosis through direct contact and newly showed that the pro-survival effect of CXCL12, BAFF and APRIL on CLL cells shown in the literature [8, 22, 23] is CLL cells/NLC contact dependant.

**Figure 2:**
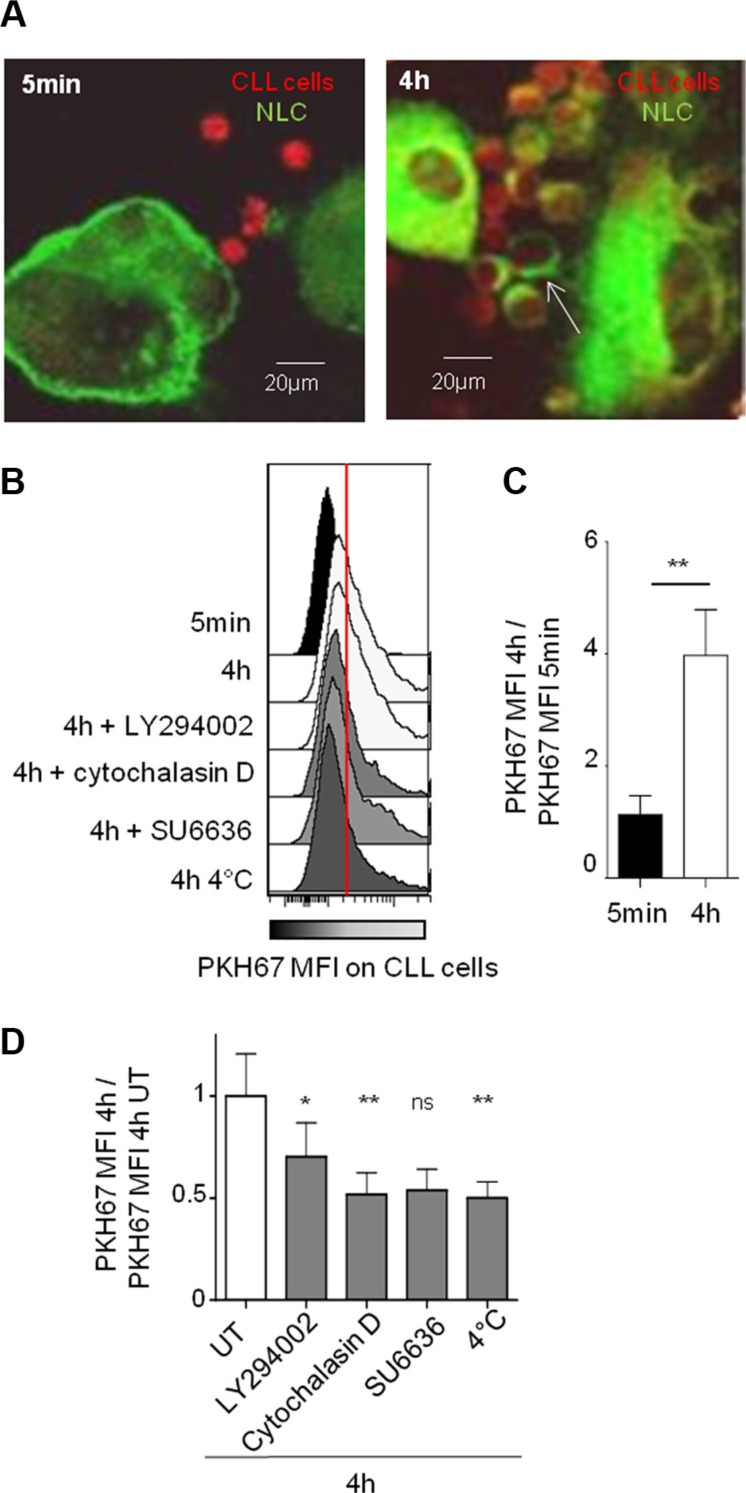
Strong and specific interactions between NLC and CLL cells are dependent on actin polymerization and on PI3K (**A**) PKH-67 (green) acquisition by CLL cells (red) after 5 min or 4 h of contact with PKH67^+^ NLC observed by confocal microscopy at 37°C. (**B**–**D**) PKH-67 acquisition by CLL cells (CD5^+^CD19^+^ gated cells) after 5 min or 4 h of contact with PKH67^+^ NLC observed by flow cytometry: one representative experiment in different conditions ((B) in the presence of different inhibitors or at 4°C), mean of 11 independent experiments at 37°C ((C) ratio of PKH67 MFI at 4 h vs PKH67 MFI at 5 min), mean of 9 independent experiments at 4°C or in the presence of different inhibitors ((D), ratio PKH67 MFI at 4 h with inhibitor treatment vs PKH67 MFI at 4 h without treatment (UT)).

To investigate the molecular mechanism underlying this protecting cell contact, we have chosen as read out trogocytosis's test. Trogocytosis is an active cell membrane transfer process induced by activating interactions between immune cells, which occurs during cell-cell contact through immunological synapses [29–31]. For this test, CLL cells were labeled with the intracellular marker CMTMR (red CMTMR^+^ CLL cells) while autologous NLC were stained with the stable PKH-67 membrane marker (green PKH67^+^ NLC). After co-culture, trogocytosis was visualized by confocal microscopy through the acquisition by red CLL cells of green membrane patches from NLC after 4 hours of contact but not after 5 minutes (Figure 2A). The validation by flow cytometry showed an increase of PKH67 MFI in all gated CLL cells after 4 hours compared to 5 minutes of contact with PKH67^+^ NLC, visualized on the Figure 2B by the transition from black (5 min) to white (4 h) (Figure 2B and 2C). Together, these results demonstrate a NLC membrane transfer to the CLL cell surface. Then, we showed that this trogocytosis was highly inhibited by low temperature, cytochalasin D (an actin polymerization inhibitor), and more weakly by LY294002, a large PI3K inhibitor (Figure 2B and 2D). Therefore, trogocytosis of NLC by CLL cells involves actin polymerization, and weakly PI3K signaling.

Altogether, our data reveal active cell contacts between CLL cells and autologous NLC, as evidenced by *in vitro* trogocytosis assays.

### LFA-3 is critical for NLC/CLL cellular cross-talk

**Figure 3:**
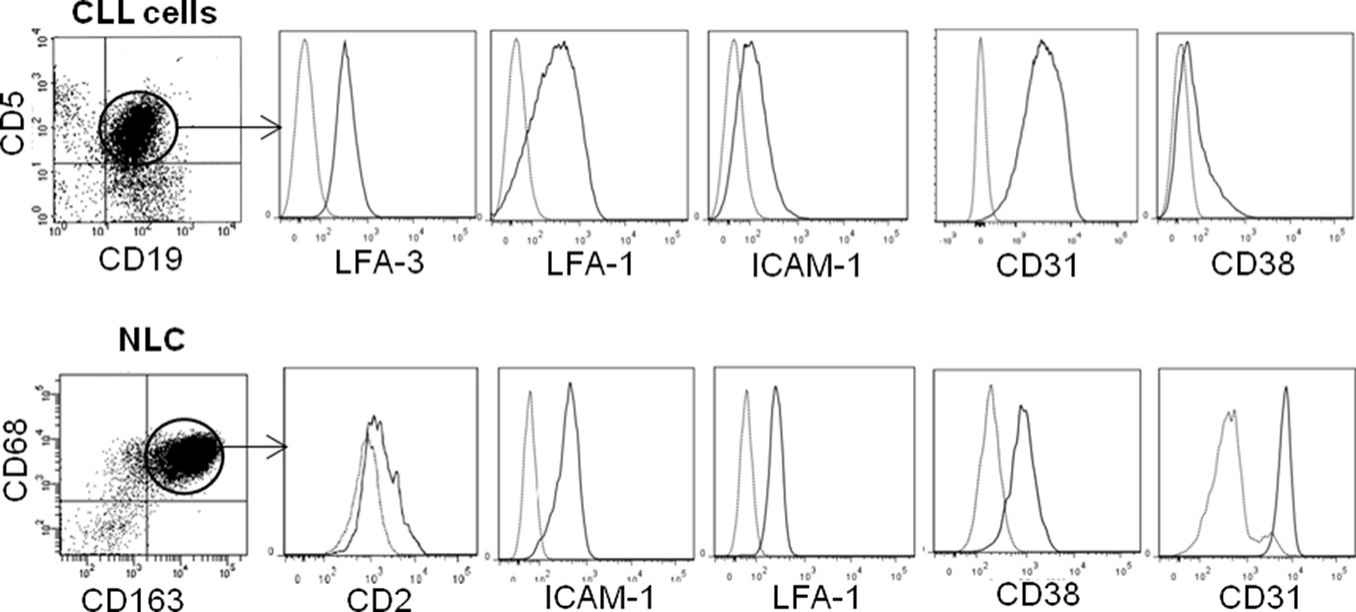
Expression of adhesion membrane proteins by NLC and CLL cells Flow cytometry analysis of adhesion molecules (black line) expressed by gated CLL cells from CLL patient's PBMC (upper) and by gated NLC from a 10 days culture with the same CLL patient's PBMC (lower), compared to isotopic control (grey line).

**Figure 4:**
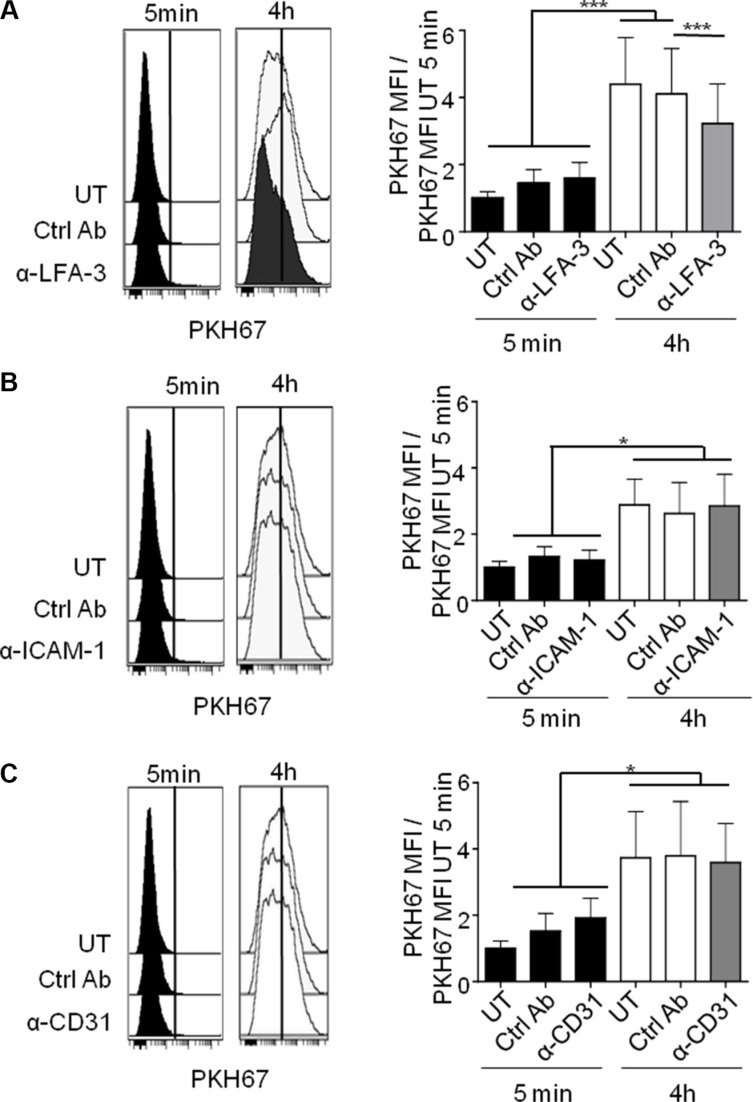
LFA-3 is involved in NLC/CLL cell contact Representative experiment (left) or cumulative histogram (right) of PKH67 acquisition by CLL cells treated or not (UT) with blocking antibodies or by their respective isotype control antibodies (ctrl Ab) after 5 min or 4 h of contact with PKH67^+^ NLC, measured by flow cytometry (ratio PKH67 MFI vs PKH67 MFI UT 5 min). (**A**) anti-LFA3 (12 CLL patients). (**B**) anti-ICAM-1 (10 CLL patients). (**C**) anti-CD31 (10 CLL patients).

**Figure 5:**
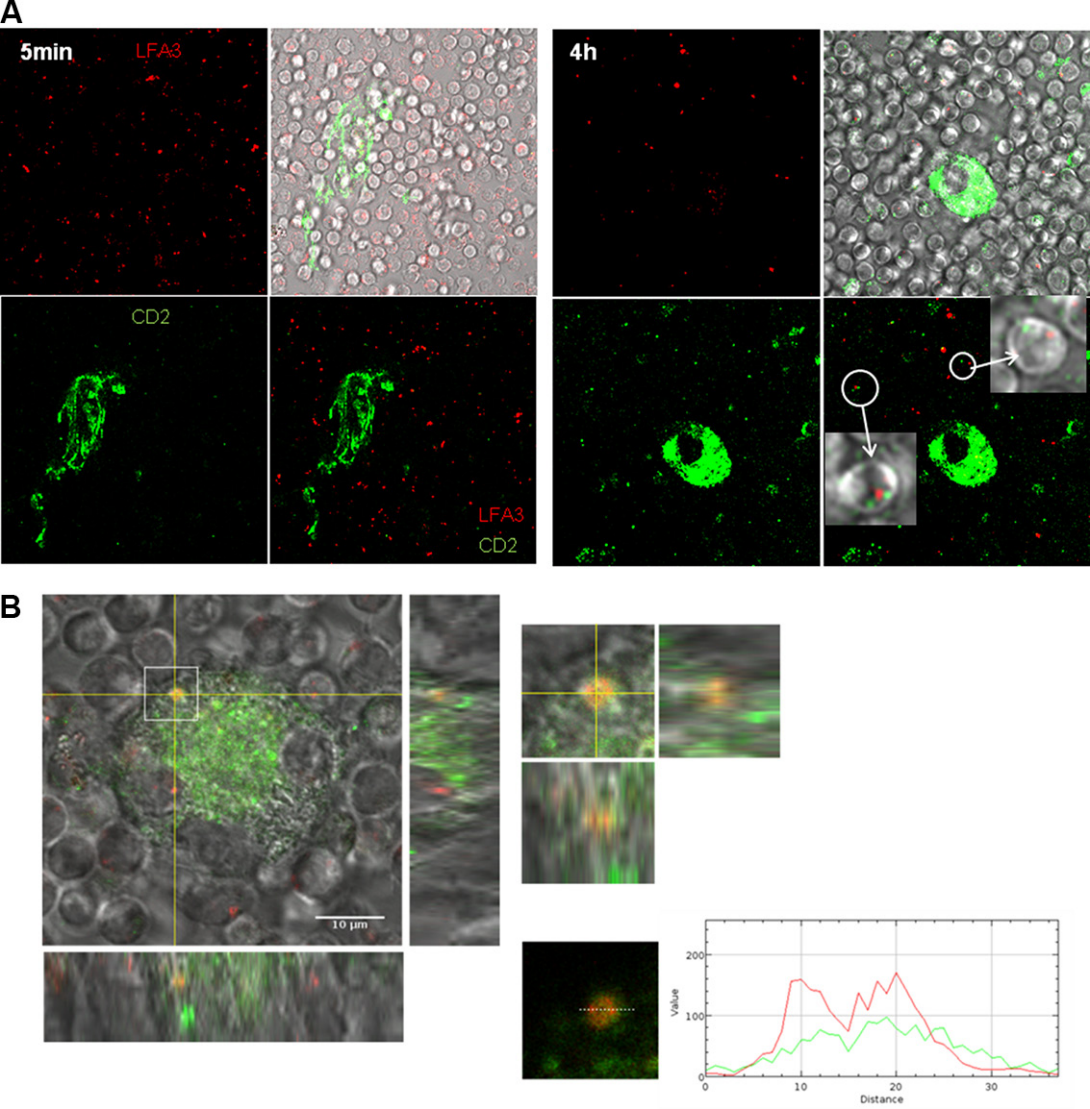
Colocalisation of CD2 and LFA-3 in the contact of NLC and CLL cell (**A**) Green CD2 acquisition by CLL cells stained by red LFA-3 after 5 min or 4 h of contact with NLC stained with green CD2 observed by confocal microscopy at 37°C (left: 5 min of contact, right: 4 h of contact). (**B**) Orange contact point characteristic of the colocalisation of green CD2 on NLC with red LFA-3 on a CLL cell placed under the NLC.

We next investigated the molecular determinants of binding between CLL cells and NLC. For this purpose, we first conducted unbiased transcriptome datamining to search for candidate molecules expressed at the cell membrane that were involved in cell binding and were overexpressed by NLC and CLL cells. Gene expression profiles (GEP) from 19 NLC (accession number: GSE87813) were compared to those of monocytes from 5 healthy donors [32]. Because we found, contrary to the literature [8, 33], that NLC, which arise from the differentiation of monocytes from CLL patients in contact with CLL cells, were protective for CLL cells but not monocytes or differentiated healthy monocytes by CLL cells [10], we chose to compare NLC with monocytes. In parallel, GEP of CLL cells from 41 patients were compared to those of 11 samples of B-lymphocyte isolated from healthy donor PBMC as depicted (downloaded from GEO dataset GSE22529) [34]. From these comparisons, we focused on genes that were significantly overexpressed (FC > 1.25, Welch corrected Student *p* < 0.05) by NLC *versus* monocytes on the one hand, and by CLL cells *versus* B-lymphocytes on the other hand. NLC overexpressed 2589 genes relative to monocytes while CLL cells overexpressed 225 genes relative to normal B-cells. These overexpressed genes were filtered out by applying functional ontology criteria such as “cell binding function” and “cell membrane expression”, which restricted these to only 27 NLC genes and 3 CLL genes (list of genes in Supplementary Table 2A and 2B). This allowed us to select the corresponding receptor/ligand couples possibly involved in the NLC/CLL cell interaction: VCAM1, CD28, CD31 (PECAM-1 gene), LFA-1 (SELPLG gene), CD2, CD86, CTLA4, CD62 (SELP gene), LFA-3 (CD58 gene) (Supplementary Table 2). Flow cytometry analysis was used to validate the cell surface expression of these proteins on the membrane of CLL cells, CD19^+^ CD5^+^ gated cells obtained from PBMC from 10 CLL patients and of NLC, CD163^+^ CD68^+^ gated cells from a 14 days culture of the same patient's PBMC. On average, we found expression of ICAM-1, LFA-1, CD38, CD31 and LFA-3 by CLL cells and expression of ICAM-1, LFA-1, CD38, CD31 and CD2 by NLC (Figure 3). From these, CD31/CD38, ICAM-1/LFA-1 and CD2/LFA-3 represented conceivable interacting candidates. To determine which of these mediated the active NLC/CLL cell contact, we used in the trogocytosis assays blocking antibodies known to block cell adhesion: anti-ICAM1 [35], anti-LFA-3 [36] and anti-CD31 [37]. Trogocytosis by CLL cells after 4 h of contact with PKH67^+^ NLC decreased significantly in the presence of blocking anti-LFA-3 compared to the control antibody (Figure 4A), while blocking ICAM-1 or CD31 had no effect (Figure 4B and 4C). Thus, LFA-3 but not ICAM-1 and CD31 is involved in NLC/CLL cell binding. To go further, we performed confocal microscopy experiments to confirm NLC/CLL cells interaction through the axis CD2/LFA-3. We co-cultured for 4 h CLL cells previously stained with red fluorescent anti-LFA-3 antibody with autologous NLC previously stained with green fluorescent anti-CD2 antibody. First, we confirmed the trogocytosis of NLC by CLL cells with the transfer of green CD2 from NLC to CLL cells after 4 h of contact (Figure 5A). Then, we showed the colocalisation of red LFA-3 and green CD2 revealed by the orange point on the confocal section of a couple of a CLL cell under a NLC (Figure 5B).

This set of experiments points towards a prominent role of the LFA-3/CD2 pair in the contact between CLL cells and NLC.

### LFA-3/CD2 interaction controls the pro-survival activity of NLC

**Figure 6:**
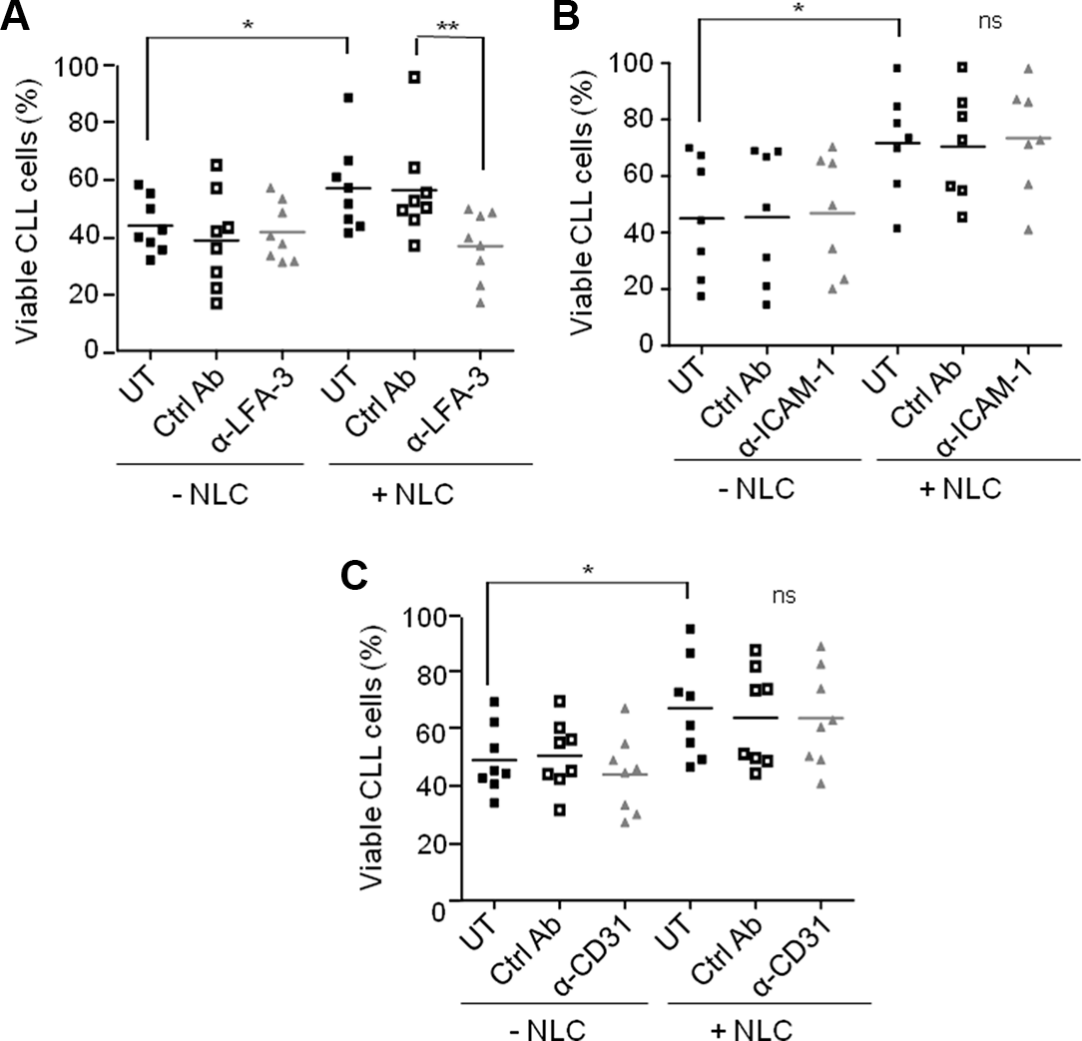
NLC protect CLL cells from apoptosis by contact through LFA-3 Percentage viability of CLL cells after 7 days of culture either alone or in the presence of NLC, treated or not (UT) with an isotype control (ctrl Ab) or with a blocking antibody: anti-LFA-3 (**A**), anti-ICAM-1 (**B**) or anti-CD31 (**C**) (8 independent experiments: 8 CLL patients).

Since NLC were involved in the protection of CLL cells from apoptosis upon contact, we asked if the receptor/ligand interaction involving LFA-3 was mandatory. CLL cell viability was tested in the presence of NLC plus a LFA-3-blocking antibody. The gain of CLL cells viability in the presence of autologous NLC (UT + NLC vs UT - NLC) was totally lost by the incubation with the anti-LFA3 blocking antibody, while the isotype control had no effect on the protective effect of NLC (Figure 6A). In contrast, neither ICAM-1 nor CD31 blocking antibodies reduced CLL cell survival regardless of the presence NLC (Figure 6B and 6C). The same decrease in CLL cells viability was obtained with anti-CD2 as with anti-LFA-3 (Supplementary Figure 1), confirming the role of the LFA-3/CD2 axis on the CLL cells viability. Control experiments validated the non-toxicity of the anti-LFA-3 blocking antibody towards B-lymphocytes, CD4 T- and CD8 T-lymphocytes from HD (healthy donor) PBMC after seven days of culture (Supplementary Figure 2).

Altogether, our data support the importance of LFA-3/CD2 molecular interactions in the pro-survival activity of NLC towards CLL cells.

### Soluble LFA-3 is released upon NLC/CLL cells interaction

**Figure 7:**
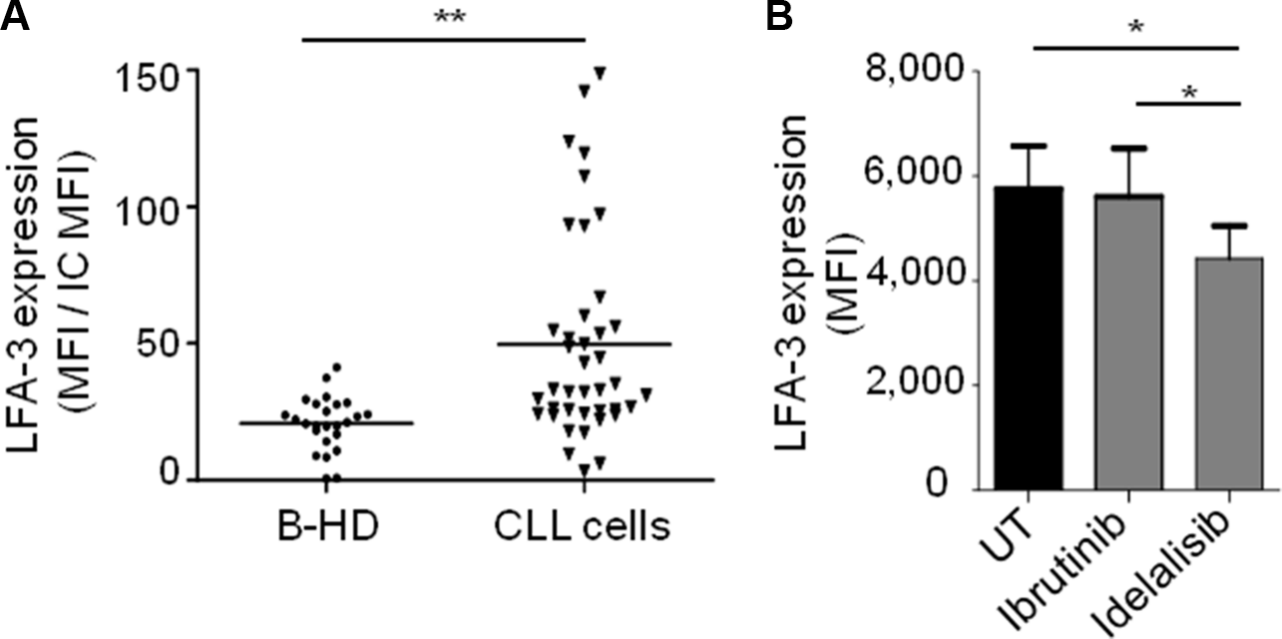
CLL cells express more of LFA-3 than healthy B lymphocytes (**A**) Expression of membrane LFA-3 by B-lymphocytes of healthy donors (B-HD, *n* = 25) and by CLL cells (*n* = 40), measured by flow cytometry (ratio: LFA-3 MFI/isotype control MFI). (**B**) Expression of membrane LFA-3 by CLL cells after 24 h of *in vitro* culture with ibrutinib (0.5 μM) or idelalisib (0.5 μM) treatment (*n* = 6).

**Figure 8:**
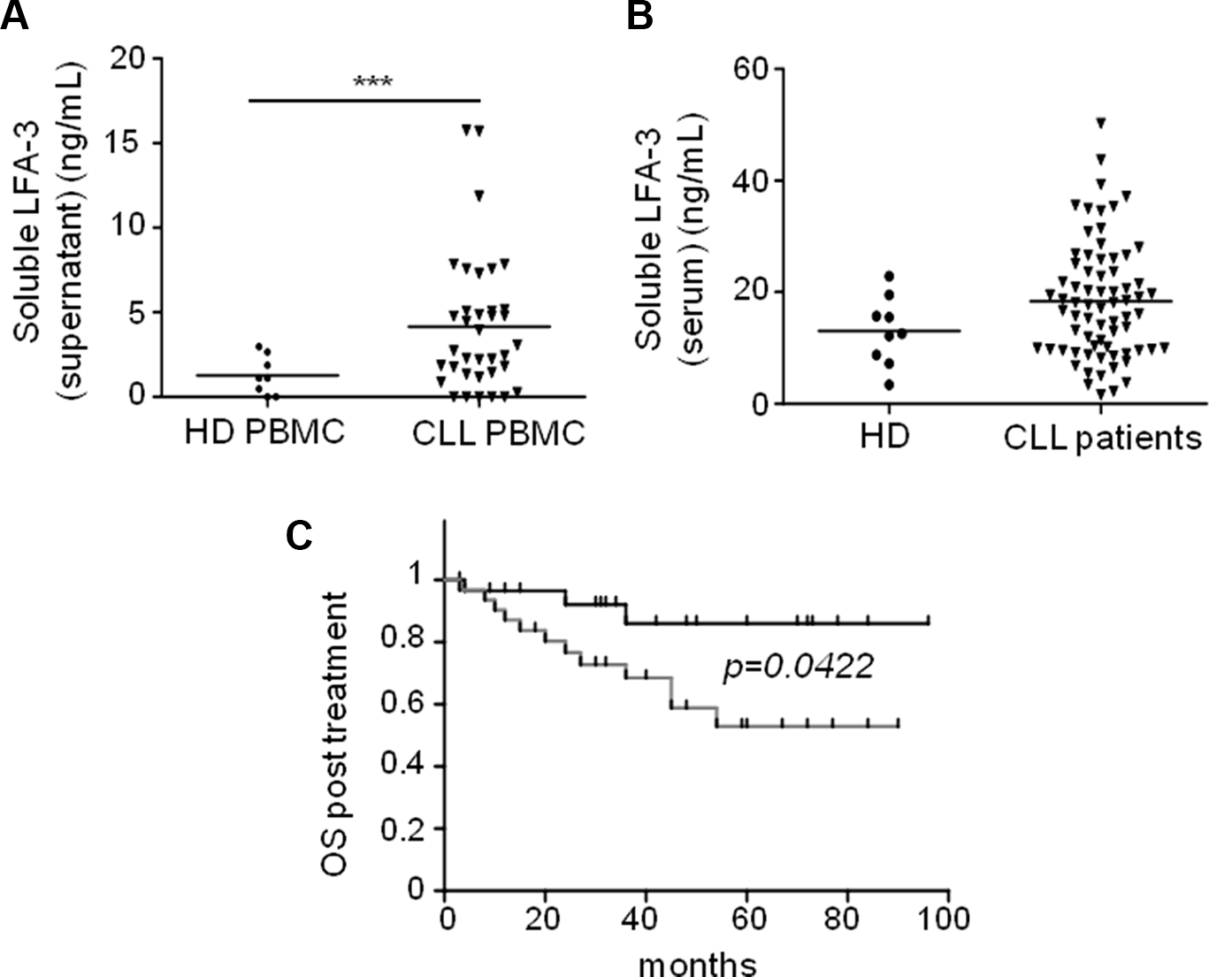
High levels of soluble LFA-3 in the serum of CLL patients are associated with poor overall survival (**A**) ELISA test of soluble LFA-3 (sLFA-3) in culture supernatants after 7 days of culture of healthy donor PBMC (*n* = 8) or CLL PBMC (*n* = 32). (**B**) ELISA test of sLFA-3 in sera from healthy donors (*n* = 9) or CLL patients (*n* = 71). (**C**) Kaplan-Meier curves showing the probability of overall survival from the first treatment. CLL patients were divided into two groups according to their sLFA-3 levels (cut-off = 16.7 ng/mL): low sLFA-3 (black) and high sLFA-3 (grey).

We could not show any correlation between CLL cell surface expression of LFA-3 and classical CLL risk factors, despite a significantly higher expression of LFA-3 in CLL cells compared to normal B-cells from healthy donors (Figure 7A). Interestingly, idelasib–but not ibrutinib–induced a decrease of LFA-3 in CLL cells (Figure 7B). Moreover, when we measured the concentration of soluble LFA-3 (sLFA-3) in PBMC cultures from CLL patients, we found it to be 3.4-fold higher than in control cultures from healthy PBMC (Figure 8A). Besides, the sLFA-3 measured by ELISA in the sera of CLL patients (*n* = 71, Supplementary Tables 3 and 4) and compared to normal donors was not found to be significantly increased (Figure 8B), in contrast to results published on other medical conditions (patients with Hodgkin's lymphomas or hepatitis [38]). However, the repartition of the sLFA-3 level for these 71 sera suggested two groups of patients: a high and a low sLFA-3 level group. Thus, to investigate any relationship between sLFA-3 levels and CLL prognosis, we searched for correlations between quantitatively high sLFA-3 expression and biological predictors of overall survival (OS) in CLL. We found a significant correlation (*p = 0.048*) between del(17p) and high levels of sLFA-3 (30.8 +/−10 ng/mL) (Supplementary Table 5). Furthermore, of the 71 patients included in our study, 60 required treatment according to the IWCLL 2008 criteria for active disease. At the time of therapy, OS post-treatment was calculated from day 1 of cycle 1. ROC analysis revealed that the best threshold of sLFA-3 discriminating OS post-treatment was 16.7 ng/mL (not shown). Accordingly, CLL patients with high levels of sLFA-3 (> 16.7 ng/mL) had a significantly shorter (*p = 0.0422*) OS post-treatment than the other patients, with survival rates at 84 months of 53% *versus* 86%, respectively (Figure 8C). This observation in a small series of course deserves further attention in much larger patient cohorts, and supports our evidence on the impact of LFA-3/CD2 interactions between NLC and CLL cells within the TME.

## DISCUSSION

Previous studies have shown that soluble factors can partially mediate NLC-induced CLL cell survival. Indeed, adding soluble CXCL12, BAFF or APRIL to purified CLL cells cultures or blocking peptides of BAFF and APRIL to CLL cells/NLC cocultures was shown to only partly increase CLL cell viability compared to that resulting from co-culture with NLC [8, 23]. Our study provides then direct evidence for the critical role of physical cellular contacts. This does not rule out a role for secreted factors which must be released consecutively to the contact between NLC and CLL cells. Indeed, CXCL12 was shown in the literature as expressed by NLC after 14 days of culture of PBMC from CLL patients [8]. If CXCL12, BAFF and APRIL had a protective effect independent of that produced by the cell contact, we would have more CLL cell death in the condition of Transwell culture plus CXCL12, BAFF and APRIL blocking. Thus, all these results possibly show a requirement for the CLL pro-survival TME.

Of the many genes encoding molecules that are potentially involved in the adhesion between tumor-associated macrophages and CLL cells, transcriptomic and phenotypic analyses indicated that the receptor/ligand pair LFA-3/CD2 was most relevant in this context. In line with reports from Tonino et al. [28], our study did not find evidence of a role for the CD38/CD31 interaction that was described by the previous Deaglio et al. report [24]. In our study, an LFA-3 blocking antibody totally inhibited the increase in survival afforded by NLC, without affecting basal survival. LFA-3/CD2 contacts may also therefore be a critical step in the release of the pro-survival soluble factors [8, 22, 23]. Notably, the LFA-3/CD2 interaction is crucial for the immune response since it stabilizes the immune synapses between T-lymphocytes and antigen presenting cells or homotypic natural killer cell cross-talk [39–41]. Our report adds a putative new function for this synaptic interaction (as shown by our trogocytosis assay), in the promotion of CLL cell survival.

LFA-3 is overexpressed by CLL cells compared to normal B-cells, so this may favor their binding to NLC or effector T-lymphocytes, although these interactions would have opposing consequences (CLL survival or lysis). However, despite the high level of CD2 expression in T-lymphocytes, the immune synapses between T-cells and tumor cells are impaired in CLL [42, 43]. Moreover, T-cell responses can be inhibited by soluble LFA-3 [38, 44–47], contributing to immunosuppression, as proposed for Hodgkin's disease [48]. Hence, we believe that LFA-3 overexpression by CLL cells promotes their pro-survival interactions with NLC rather than anti-tumoral immunity.

The shedding of soluble LFA-3, a phospholipase by-product of cell surface LFA-3 [44], into both culture medium and human sera, has been previously reported in patients with rheumatoid arthritis, spondylarthropathies, liver cirrhosis, hepatocellular carcinomas, Hodgkin's disease, multiple myeloma and non-Hodgkin's lymphoma [38, 47], so our report now adds CLL to this list. Although soluble LFA-3 levels are higher in patients with liver cirrhosis and hepatocellular carcinomas [38, 47] than in healthy donors, they remain globally similar to what has been measured for hematological malignancies [38], including our 41 CLL patients. Nevertheless, we suggest that high levels of sLFA-3 may correlate with prognosis and provide another critical determinant of OS (such as del(17p)), consistent with the published adverse prognosis for patients based on HMGB1 expression and NLC/CLL interactions [21]. We could imagine that high blood level of sLFA-3 favors a defective CLL cells/T cells synapse as CD2 is involved as an accessory molecule in the T cell receptor signaling [39]. However, as NLC are only located in TME [49], nursing effect of NLC for CLL cells in TME would not be impaired by sLFA-3, favouring the disease progression. We also propose that idelalisib therapy, even in the frontline setting, may specifically alleviate this detrimental effect of NLC/CLL in the TME through decreased LFA-3 expression in CLL cells, an effect that was not observed with ibrutinib *in vitro*. Accordingly, we recently published data suggesting that idelalisib alone is more effective than ibrutinib (at the same clinically-relevant dose of 0.5 μM) in combating the pro-survival effects of NLC [49].

Our demonstration that LFA-3 blockade inhibits the pro-tumoral activity of NLC *in vitro* and does not affect the viability of non-malignant lymphocytes posits this as a candidate target for CLL therapy. Nevertheless, its widespread expression by most hematological cell types raises issues on the benefits/risks balance. Indeed, other therapeutic monoclonal antibodies raised against widely expressed antigens induced limiting toxicities which ultimately led to their withdrawal. For this case, PI3K/AKT inhibitors may then be used with a view of neutralizing NLC-derived signals to CLL cells.

Despite this, our results begin to unravel the mechanisms underlying the NLC-CLL cell relationship which paves the way for targeting this pro-survival process in the future.

## MATERIALS AND METHODS

### Cell preparation and culture

Blood samples were obtained after informed consent and stored at the HIMIP collection. According to French law, the HIMIP collection was declared to the Ministry of Higher Education and Research (DC 2008-307 collection 1) and a transfer agreement (AC 2008-129) was agreed after approbation by the “Comité de Protection des Personnes Sud-Ouest et Outremer II” (ethical committee). Clinical and biological annotation of samples were declared to the Comité National Informatique et Libertés (CNIL; data processing and liberties national committee).

PBMC were isolated by density-gradient centrifugation from blood samples of CLL patients or healthy donors (HD). To generate NLC, PBMC from CLL patients were cultured (10^7^/ml) as previously described [8]. Before each experiment, CLL cells were separated from NLC by vigorous pipetting (the purity of CLL cells and NLC was checked by flow cytometry, greater than 95%).

### Determination of IGHV status, FISH abnormalities and recurrent mutations

The mutation hotspots of the *TP53* (exons 4-9; RefSeq NM_000546.5), *SF3B1* (exons 14, 15, 16; RefSeq NM_012433.2) and *NOTCH1* (exon 34; RefSeq NM_017617.2) genes were screened by polymerase chain reaction (PCR), followed by high resolution fusion in 10 μl on a LightCycler LC 480 (Roche, Basel, Switzerland). For *TP53* and *SF3B1*, recurrent mutations were validated by Sanger sequencing (3130 XL Genetic Analyzer, Applied Biosystems, Foster City, CA) of positive amplicons. The recurrent mutation delCT of *NOTCH1* (c.7544_7545delCT) was validated by capillary electrophoresis of amplicons. *TP53* mutations and/or *TP53* deletion were pooled together as *TP53* alterations. (primers: Supplementary Table 1).

### CLL cell viability

After 14 days of culture of CLL patient's PBMC, CLL cells was separated from generated adherent NLC (purity greater than 95%). These purified CLL cells were again co-cultured for 7 days with the autologous NLC with or without a Transwell membrane with 0.4 μm pores (Corning, NY, USA), CLL cells being in the Transwell insert, and with or without drugs or blocking antibodies. Viability of CLL cells was measured after 7 days of co-culture with the ADAM-automatic fluorescent cell counter based on the propidium iodure staining.

### Reagents and blocking antibodies

Cytochalasin D, SU6656 and LY-294002 (Sigma-Aldrich, St. Louis, MO, USA) were used at 10 μM, 1 μM and 1 μM, respectively. Blocking antibodies anti-CD2 (clone TS2/18), anti-CD31 (clone HEC7), anti-ICAM-1 (clone W-CAM-1), anti-LFA-3 (clone TS2/9) and their relevant isotype controls were used at 10 μg/ml (Thermofisher, Villebon sur Yvette, France). Blocking antibodies anti-CXCL12 (Selleckchem, Houston, USA), anti-BAFF and anti-APRIL (clone 670820, R&D Systems, Minneapolis, USA) were used at 4 μM, 20 ng/ml and 1 μg/ml respectively.

### Flow cytometry

Cells were labeled with 5 μg/ml antibodies or isotype controls for 20 minutes at 4°C and analyzed on an LSRII cytometer (BD Biosciences, Pont de Claix, France). Data were analyzed using BD FACSDiva software, FlowJo software or Cytobank software (https://www.cytobank.org/cytobank/).

We used: PC7 anti-CD5, APC-Cy7 anti-CD14, APC anti-CD163, FITC anti-CD2 and isotype controls (Ozyme, France); APC-Cy7 anti-CD19, Alexa-fluor 647 anti-CD31, PE anti-LFA-1 and isotype controls (Pharmigen, France); PE anti-CD38, PE anti-ICAM-1, PE anti-LFA-3 and isotype controls (Beckman Coulter, Fullerton, CA, USA); APC-Cy7 anti-CD3 and isotype control (Tonbo Biosciences, San Diego, CA, USA).

### Trogocytosis experiments

PBMC from CLL patients were cultured for 15 days on a LAB TECH II Chamber Slide (NUNC, NY, USA), at 10^6^/ml in 500 μL. CLL cells were discarded then stained with the CellTracker Orange CMTMR (5-(and-6)-(((4-chloromethyl)benzoyl)amino)tetramethylrhodamine) according to the manufacturer's instructions (Sigma-Aldrich, St. Louis, MO, USA) or with an anti-LFA-3 (clone 2D11-B10, Abnova, Montluçon, France) and a goat anti mouse coupled to alexa fluor 633. Autologous adherent NLC were stained with PKH67 according to the manufacturer's instructions (Sigma-Aldrich, St. Louis, MO, USA) or with an anti-CD2 (clone EPR3693, Abcam, Cambridge, England) and with a goat anti rabbit coupled to alexa fluor 488. NLC and CLL cells were co-cultured (0.4 × 10^6^) for 5 minutes or 4 hours at 37°C with or without inhibitors or blocking antibodies, or at 4°C. Cells were analyzed by flow cytometry or fixed with PBS 4% paraformaldehyde, then washed and mounted in PBS containing 90% glycerol and 2% 1-4-diazabicyclooctane (DABCO; Sigma-Aldrich, St. Louis, MO, USA), before being examined using a Carl Zeiss LSM 710 confocal microscope (Carl Zeiss, Mary le Roi, France).

### Gene expression analysis and data mining

NLC from 19 CLL patients were obtained by the culture of their PBMC for 14 days, then purified by elimination of the supernatant followed by three washing with PBS (> 95% purity). Total RNA was extracted of about 1 × 10^6^ NLC using TRIzolTM reagent (Invitrogen), according to the manufacturer's instructions. The quality and integrity of the RNA obtained were assessed by using an Agilent 2100 Bioanalyzer (Agilent Technologies, Santa Clara, CA, USA) after a denaturating step at 70°C for 2 min. cRNA was prepared according to One-Cycle Target Labeling protocol (Affymetrix, Santa Clara, CA, USA) starting from 1 μg total RNA. cRNA was fragmented and hybridized to Affymetrix HG-U133 plus 2.0 arrays. The chips were then washed and scanned, according to the manufacturer's instructions.

Raw data (Affymetrix CEL files, HG U133-Plus 2.0 platform) from the 19 purified NLC and from 5 samples of CD14^+^ monocytes from healthy individuals [32], were together normalized in batches with the RMA software. Geo accession number is GSE87813. The 54,676 probe sets were then reduced to a total of 20,606 genes (HUGO symbols) using the GSEA's collapse function set on maximal probe mode (GSEA, http://www.broadinstitute.org/gsea). The expression levels of adhesion molecules were analyzed after log (base 2) transformation, normalization and collapse, from KEGG lists (http://www.genome.jp/kegg/) describing adhesion phenomenon (adherent junctions, cell adhesion molecules, focal adhesion, gap junctions and tight junctions). We then restricted the genes to “cell binding function” and “cell membrane expression”. Then, 52 additional raw data files of purified B cells from 41 untreated CLL patients and 11 age-matched control subjects (GSE22529) [34] were treated as above.

### Serum collection and ELISA

Blood samples were collected then performed using BD Vacutainers (BD Biosciences) to collect serum. Supernatants of HD PBMC or CLL PBMC cultures were obtained by centrifugation (500 g, 10 minutes). ELISA test was used to identify soluble LFA-3 (sLFA-3) (Cusabio, Wuhan, Hubei Province, China).

### Statistics

Data shown represent means ± SEM with *p < 0.05, **p < 0.01, ***p < 0.005. Data from transcriptomic analysis were analyzed using a two-tailed, unpaired and Welch corrected Student's *t*-test. Cell viability, trogocytosis experiments and LFA-3 expression was analyzed using a Wilcoxon matched paired test.

The overall survival (OS) post-treatment was defined as the time from the first treatment to the date of death if patients died or were censored on the last visit. OS post-treatment was estimated by the Kaplan-Meier method and assessed by the log-rank test.

## SUPPLEMENTARY FIGURES AND TABLES


