# Supplementary material for: Nurse-like cells promote CLL survival through LFA-3/CD2 interactions
Source: Oncotarget. 2016 Nov 26;8(32):52225–36. doi: 10.18632/oncotarget.13660 (PMC5581024; doi:10.18632/oncotarget.13660)
Supplement: Supplementary file 1 [file oncotarget-08-52225-s001.pdf]

Nurse-like cells promote CLL survival through LFA-3/CD2 interactions

Supplementary Materials

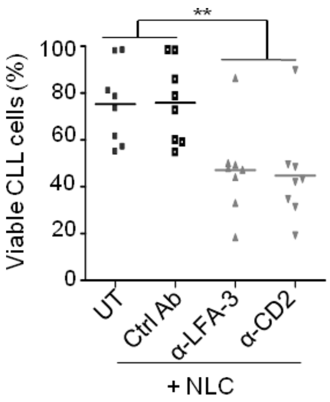

Supplementary Figure 1: Decrease of the CLL viability by an anti-CD2 blocking antibody. Percentage viability of CLL cells after 7 days of culture with NLC treated or not (UT) with blocking antibody (anti-LFA-3 or anti-CD2) or the respective control antibody (8 independent experiments).

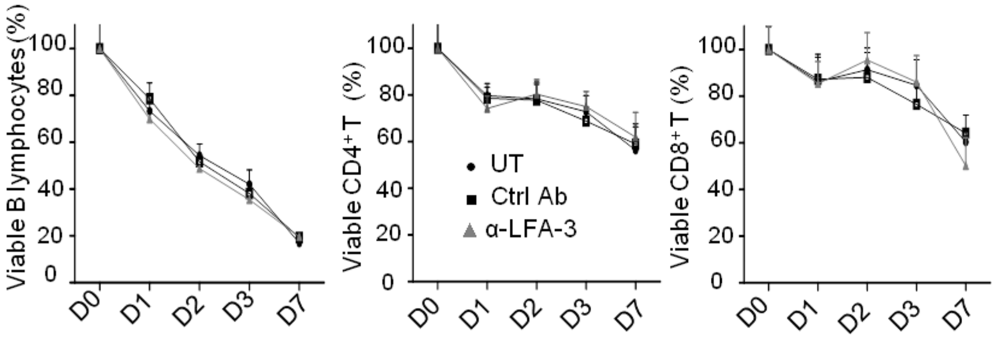

Supplementary Figure 2: Non-toxicity of the anti-LFA-3 blocking antibody towards healthy lymphocytes. Percentage viability of B-lymphocytes (left), T CD4^{+} cell (center) or T CD8^{+} cells (right) after 7 days of culture treated or not (UT) with blocking anti-LFA-3 or the control antibody (4 independent experiments: 4 healthy donors).

Supplementary Table 1: Details of primers (S: sense, AS: antisense)

Gene	Exon	Details
TP53	Exon 4	S : CTGCACCAGCAGCTCCTACA<br>AS : CAGGCATTGAAGTCTCATGGAA
	Exon 5	S : CAACTCTGTCTCCTTCCTCTTCCTAC<br>AS : GTCGTCTCTCCAGCCCCAGC
	Exon 6	S : CCTCTGATTCCTCACTGATTGC<br>AS : CTTAACCCCTCCTCCCAGAG
	Exon 7	S : CCTCATCTTGGGCCTGTGTTA<br>AS : GCTTCTTGTCCTGCTTGCTT
	Exon 8	S : TTGCTTCTCTTTTCCTATCCTGA<br>AS : GCTTCTTGTCCTGCTTGCTT
	Exon 9	S : CCTTTCCTTGCCTCTTTCCT<br>AS : CCACTTGATAAGAGGTCCCAAG
	Exon 10	S : TCCCCCTCCTCTGTTGCT<br>AS : GAATCCTATGGCTTTCCAACCTAG
SF3B1	Exon 14	S : GTCTGGCTACTATGATCTCTACCATGA<br>AS : GATGTGGCAAGATGGCACAG
	Exon 15	S : TGAATAGTTGATATATTGAGAGAATCTGGA<br>AS : AGGTAATTGGTGGATTTACCTTTCC
	Exon 16	S : TGTGTTAAAGCCTTTATGGAAGGGTAT<br>AS : ACATATCCAGTTTACATTAACAAATCTGGA
NOTCH1	Exon 34	S : GTGACCGCAGCCCAGTTC<br>AS : GGCGATCTGGGACTGCAT

Supplementary Table 2: Upregulated genes in NLC (19 CLL patients) compared to HD monocytes (5 healthy donors) (A) or in CLL cells (41 CLL patients) compared to B–HD (11 healthy donors) (B) involved in the adhesion phenomenon

A

Symbol	Description	Fold change (log(2))	*p* value
**HTR2B**	HTR2B:5-hydroxytryptamine (serotonin) receptor 2B	1.82	1.45E-05
**HLA-DMB**	HLA-DMB:major histocompatibility complex, class II, DM beta	1.76	1.02E-05
**VCAM1**	VCAM1:vascular cell adhesion molecule 1	1.72	9.74E-05
**VCL**	VCL:vinculin	1.65	2.53E-07
**HLA-DMA**	HLA-DMA:major histocompatibility complex, class II, DM alpha	1.60	3.02E-04
**ITGB5**	ITGB5:integrin, beta 5	1.59	2.09E-05
**ITGA6**	ITGA6:integrin, alpha 6	1.57	5.71E-05
**CD28**	CD28:CD28 molecule	1.52	7.14E-05
**CD276**	CD276:CD276 molecule	1.48	1.06E-07
**ITGAM**	ITGAM:integrin, alpha M (complement component 3 receptor 3 subunit)	1.48	2.59E-04
**F11R**	F11R:F11 receptor	1.47	4.90E-06
**PECAM1**	PECAM1:platelet/endothelial cell adhesion molecule (CD31 antigen)	1.47	1.51E-05
**ITGB2**	ITGB2:integrin, beta 2 (complement component 3 receptor 3 and 4 subunit)	1.47	1.40E-03
**SELPLG**	SELPLG:selectin P ligand	1.38	8.93E-08
**EPB41**	EPB41:erythrocyte membrane protein band 4.1 (elliptocytosis 1, RH-linked)	1.36	1.14E-04
**ITGA4**	ITGA4:integrin, alpha 4 (antigen CD49D, alpha 4 subunit of VLA-4 receptor)	1.34	2.14E-03
**HLA-DQB1**	HLA-DQB1:major histocompatibility complex, class II, DQ beta 1	1.34	6.76E-03
**CD2**	CD2:CD2 molecule	1.32	4.72E-04
**ITGA3**	ITGA3:integrin, alpha 3 (antigen CD49C, alpha 3 subunit of VLA-3 receptor)	1.31	1.03E-04
**CD86**	CD86:CD86 molecule	1.30	5.62E-04
**HLA-DPB1**	HLA-DPB1:major histocompatibility complex, class II, DP beta 1	1.30	1.03E-03
**HLA-DOA**	HLA-DOA:major histocompatibility complex, class II, DO alpha	1.30	9.43E-07
**HLA-DRB4**	HLA-DRB4:major histocompatibility complex, class II, DR beta 4	1.29	1.73E-02
**HLA-DQA1**	HLA-DQA1:major histocompatibility complex, class II, DQ alpha 1	1.27	2.46E-03
**HLA-DPA1**	HLA-DPA1:major histocompatibility complex, class II, DP alpha 1	1.27	2.41E-02

B

Symbol	Description	Fold change (log(2))	*p* value
**CTLA4**	CTLA4:cytotoxic T-lymphocyte-associated protein 4	1.89	8.38E-06
**SELP**	SELP:selectin P (granule membrane protein 140kDa, antigen CD62)	1.32	9.38E-06
**CD58**	CD58:CD58 molecule	1.28	4.98E-03

Supplementary Table 3: Clinical characteristics of 71 patients with CLL: categorical variables (A) and continuous variables (B)

A

**Characteristics**	**Category**	***n* (%)**
Sex	Female	21 (29.6)
	Male	50 (70.4)
IgHV status	Mutated	29 (46.0)
	Unmutated	34 (54)
Cytogenetics	Tri 12	9 (12.6)
	Del(13q)	21 -30.6)
	Del(6q)	3 (4.2)
	t(BCL2)	3 (4.2)
	Del(11q)	8 (11.2)
	Del(17p)	3 (4.2)
	Complex karyotype	13 -18.3)
Recurrents mutations	*SF3B1*	3 (7)
	NOTCH1	11 (15.4)
	TP53	6 (8.4)

B

**Characteristics**	**Mean (SD)**	**Median (range)**
Age	66.3 (9.00)	66 (41-86)
Lymphocytosis (G/L)	47.89 (33.40)	45.8 (6.5–113.8)
Monocytosis (G/L)	1.30 (1.01)	1.02 (0–4.2)

Del: deletion, IgHV: immunoglobulin heavy chain variable segment mutational status, tri 12: trisomy 12.

Supplementary Table 4: Overall survival in the different prognostic subgroups

Characteristics	Category	Total	Patients death	*p*
	All patients	71	15	-
Binet Stage	A	27	5	-
	B	21	4	0.7201
	C	19	4	0.9024
IgHV status	Mutated	29	4	-
	Unmutated	34	9	0.1013
Cytogenetics	Tri 12	9	2	0.9001
	Del (13q)	21	6	0.2993
	Del (6q)	3	1	0.7979
	t (BCL2)	3	0	0.4794
	Del (11q)	8	2	0.259
	Del (17p)	3	2	0.008
	Complex karyotype	13	3	0.7728
Recurrents mutations	*SF3B1*	3	1	0.8503
	NOTCH1	11	2	0.7367
	TP53	6	3	0.1183

In our series, only del (17p) were correlated with shorter overall survival post treatment. Del: deletion, IgHV: immunoglobulin heavy chain variable segment mutational status, tri 12: trisomy 12.

Supplementary Table 5: Level and correlation between high sLFA-3 (> 16.7 ng/mL) and established prognostics markers in CLL

Characteristics	Category	Median sLFA–3 (range)	p sLFA-3
	All patients	17.72 (1.65–50.26)	-
Binet stage	A	15.73 (1.65–50.26)	-
	B	18.11 (2.24–43.71)	
	C	17.12 (3.83–50.26)	0.6704
Age	< 65y	12.46 (3.43–50.26)	-
	> 65y	18.12(1.65–43.71)	0.1229
IgHV status	Mutated	17.12 (3.43–39.35)	-
	Unmutated	19.21 (2.24–50.26)	0.3545
Cytogenetics	Tri 12	9.58 (2.24–34.98)	0.1134
	Del (13q)	17.72 (3.43–50.26)	0.3011
	Del (6q)	20.10 (8.99–26.85)	0.8217
	t (BCL2)	10.18 (2.24–50.26)	0.5397
	Del (11q)	18.12 (12.96–50.26)	0.2163
	Del (17p)	30.81 (20.69–34.98)	0.0478
	Complex karyotype	18.71 (2.24–50.26)	0.6464
Recurrents mutations	*SF3B1*	18.12 (13.75–50.26)	0.4315
	NOTCH1	25.06 (2.24–37.16)	0.5662
	TP53	18.9 (13.15–30.81)	0.37

In our series, only del (17p) were correlated with high sLFA-3 levels. Del: deletion, IgHV: immunoglobulin heavy chain variable segment mutational status, tri 12: trisomy 12. Comparison of two groups (for example Tri 12: present or not) was made using Mann-Whitney test.
